# Assessing the relationship between domestic work experience and musculoskeletal health among rural Nigerian women

**DOI:** 10.1371/journal.pone.0276380

**Published:** 2022-12-13

**Authors:** Abisola Osinuga, Nathan B. Fethke, William T. Story, Segun E. Ibitoye, Kelly K. Baker

**Affiliations:** 1 Department of Occupational and Environmental Health, College of Public Health, University of Iowa, Iowa City, Iowa, United States of America; 2 Department of Community and Behavioral Health, College of Public Health, University of Iowa, Iowa City, IowaUnited States of America; 3 Department of Health Promotion and Education, University of Ibadan, Ibadan, Nigeria; University of Uyo, NIGERIA

## Abstract

**Background:**

Women performing strenuous domestic tasks (especially those in developing countries) are at risk of experiencing musculoskeletal pain (MSP). Physical, psychosocial, and social conditions of work in rural environments contribute to women’s domestic work experiences (DWEs) and the risk of MSP. The impact of DWEs on women’s health is especially severe in water-insecure countries like Nigeria. This study examines the relationship between a recently developed measure of DWEs and self-reported pain in the lower back (LBP), neck/shoulder (NSP), and elbow/hand/wrist regions (EHWP) among rural Nigerian women.

**Methods:**

Interviewer-administered survey data were collected from 356 women in four rural communities of Ibadan, Nigeria. Binary and ordinal logistic regression models were used to examine the relationship between DWE factor scores, sociodemographic characteristics, and musculoskeletal pain symptoms and severity after controlling for sociodemographic covariates. Effect estimates of association were presented using the odds ratio (OR), and the corresponding 95% confidence interval (CI) at *p*-value of 0.05.

**Findings:**

Among 356 participants, the 2-month prevalence of LBP was 58%, NSP was 30%, and EWHP 30%. High DWE scores were significantly associated with higher odds of experiencing and having more severe LBP, NSP, and EHWP. Specifically, the odds of LBP [(OR = 2.88; 95% CI = 1.64–5.11), NSP (OR = 4.58; 95% CI = 2.29–9.40) and EHWP (OR = 1.88; 95% CI = 1.26–3.77)] were significantly higher among women who perceived their domestic work responsibilities as very stressful (i.e., ‘high stress appraisal’) compared to those with lower stress appraisal scores. Those who were time-pressured and had less autonomy over familial duties (i.e., ‘high demand/low control’) had significantly higher odds of LBP [(OR = 2.58; 95% CI = 1.64–4.09) and NSP (OR = 1.49; 95% CI = 1.24–2.58)]. Frequently fetching and carrying water over long distances and time (i.e., ‘high water sourcing and carriage’) was also associated with higher odds of LBP [(OR = 1.31; 95% CI = 1.09–1.79) and NSP (OR = 1.20; 95% CI = 1.08–1.76).

**Conclusion:**

Strenuous and stressful DWEs were associated with MSP among rural Nigerian women. This study provides new evidence on how the physical, social, and psychosocial factors of domestic work can increase women’s risk of MSP.

## 1. Introduction

Musculoskeletal disorders (MSDs) are the leading contributor to disability and years lived with disabilities (YLDs), responsible for 17% of YLDs in 160 countries [1]. MSDs may be localized or involve multiple body areas or systems [2,3]. They are typically characterized by pain that may be intermittent, of varying intensity, persistent [4], debilitating, and functionally limiting, resulting in loss of time from work or reduced work productivity [1,5,6]. According to multiple Global Burden of Disease Studies performed in the past three decades, low back pain (LBP) (the main contributor to MSD burden) and neck pain remain the leading causes of disability-adjusted life years (DALYs) and YLDs, globally [7–10]. Individual and lifestyle factors (gender, age, obesity, smoking) [11–15], work-related physical factors [16–18], and psychosocial factors [3,19–22] have been reported to contribute to MSDs from population-based and occupational studies. In LMICs such as Nigeria, research on the domestic or paid work-related physical and psychosocial risk factors for MSDs is limited compared to high-income countries (HICs).

Globally, in the general population and across occupations, women tend to have a greater prevalence of MSDs than men [23,24]. For instance, the age-standardized point prevalence of neck pain and back pain from 1990–2017 was higher among females (8.05%) than males (6.94%) in Global Burden of Diseases studies [9,10]. The gendered difference in MSD prevalence has been attributed to differential exposure to physical risk factors during paid and unpaid work. In some occupational studies, for example, the greater observed MSDs risk of neck/shoulder pain among women compared to men has been attributed clustering of women in service-based occupations that require repetitive motions, static work postures, and manual material handling [25–30]. In addition, some occupational studies have evaluated gender differences between psychosocial work variables (such as job demand/control and level of social support at work) and MSDs. Those studies observed that women had a higher risk than men even when work tasks were identical [3,31]. Other studies have observed no significant difference in the prevalence or risk of MSDs due to psychosocial work factors [25,29]. The factors contributing to gendered differences in the prevalence or risk of MSDs have not been thoroughly studied. The physical demands of domestic work have been associated with LBP from studies in HICs and LMICs [27,32,33]. The physical, psychosocial, and social conditions of domestic work may be critical causal factors contributing to increased MSD prevalence among women. Understanding the relationship between conditions of domestic work and MSD among women is particularly important in LMICs, where the physical demands have been associated with high risks of back pain (a common MSD) [32].

### 1.1. Domestic work experiences and musculoskeletal pain

Millions of women in Asia and Africa spend approximately half of their day performing strenuous domestic tasks [34,35]. From our previous work on characterizing the domestic work experience (DWE) of rural Nigerian women, women spend approximately 30 hours per week on domestic tasks such as cooking, childcare, water fetching, and manual dish/clothes washing [36]. Physically demanding domestic work is associated with a higher risk of back pain in studies from LMICs compared to studies from higher-income countries [32]. The physical, psychosocial, and social conditions in which domestic tasks are performed may increase women’s risk of musculoskeletal pain (MSP). DWE measures are latent variables that measure the physical, social, and psychosocial conditions of women’s work. Frequent engagement in multiple household responsibilities, including childcare, has been associated with the risk of MSP [37].

The experience of water scarcity and labor associated with water collection also contributes to a woman’s DWE [36]. In Nigeria, more than two-thirds of women rely on dug-wells (68%) and transport water via head loading [36]. Head porterage could increase the pressure on the neck (compression forces), particularly if the load is heavy, and may increase the risk of neck or back pain [38,39]. Thus, the risk of MSP may be disproportionately high for women living in water-insecure countries, like Nigeria, which has the second highest number of women who spend more than 30 minutes collecting water among 24 Sub-Saharan African countries [40]. Other domestic tasks, such as child carrying/caregiving, manual laundry, and food preparation/processing activities, may involve heavy lifting, pounding, grinding, manual handling, and repetitive motions, which could increase MSP risk [41,42].

While self-reported pain was identified as a health outcome of specific domestic tasks such as fuel and water carriage in reviews [32,43], examining the health risks from these specific domestic tasks alone as it has been done in the literature presents a limited scope of the burden and consequences of domestic labor, especially in developing countries. In addition, studies on the associations between social and psychosocial factors, such as job demands, decision authority, low control, and psychosocial stress with musculoskeletal pain, have been primarily from occupational/paid work settings. A review of occupational studies found weak associations between high work demands and low control at work as risk factors for neck/shoulder pain [12]. Another systematic review and meta-analysis of 18 studies found that high job control, decision authority, and social support were protective against chronic LBP [44]. Few studies have evaluated gender-specific relationships between psychosocial work variables and musculoskeletal pain, even within the occupational sector. Given that physical, psychosocial, and social conditions of work have been associated with MSP in occupational settings, evaluating how these conditions in domestic work impact women’s risk of MSP in LMICs could improve knowledge of risk factors contributing to the global burden of MSDs. This study addresses a significant knowledge gap on the broader relationships between MSP and the psychosocial stress from domestic work demands, the physical stress of water carriage, and other domestic tasks in a low-income, water-insecure setting (rural Nigerian women).

## 2. Methods

### 2.1. Study setting and survey design

A cross sectional study design was used to understand the association between the physical, psychosocial, and social factors of DWE and MSP in the lower back, neck/shoulder, and elbow/wrist/hand among rural Nigerian women. Women of reproductive ages (18–49 years) were recruited from four neighboring rural communities in Lagelu and Akinyele Local Government areas, Oyo State, Nigeria. Eligible participants were recruited by interviewers (graduate students at the University of Ibadan) using a systematic sampling procedure. Every fifth house on the streets in the communities was visited to introduce the research and ascertain eligibility by asking three main questions: frequency of engagement in domestic work, age of the woman responsible for domestic work responsibilities, and presence of any chronic illness.

Informed consent was received from the participants. Copies translated to Yoruba were provided to and read to illiterate/semi-literate participants in the presence of a witness before consent was obtained. Translated survey copies were also provided. Interviewers administered (face-to-face) the survey to the participants. Full details of the survey development and administration process, DWE final measures, and the finalized survey instrument are described in a previous paper [36].

#### 2.1.1 Ethical review

The study was approved by the Ethics Review Committee of the Oyo State Ministry of Health, Planning, Research and Statistics Department (approval ID:AD/13/479/223) and by the University of Iowa International Review Board (IRB ID: 201904718).

### 2.2. Measures

Independent Variables: Primary independent variables included factor scores from six DWE measures (latent factors derived from confirmatory factor analysis of responses to survey questions [36]) and self-reported total hours spent on domestic work per week [recoded into *lowest* (all hours equal to or less than the 1^st^ quartile -25th percentile value); *middle* quartile (all hours that are greater than the 25 percentile value but less than the 75^th^ percentile value); and *highest* quartile (all hours equal to or greater than 75% percentile value) hours of domestic work). The six DWE measures were divided into three domains: physical (frequency of domestic work, water sourcing and carriage, and experience of water scarcity), psychosocial (stress appraisal and demand-control), and social (social support). Details on the measure development process were discussed in a previous article [36].

Sociodemographic characteristics such as age, household income [recoded into *lowest* (all income values equal to or less than the 1^st^ quartile -25th percentile value); *middle* quartile (all income values that are greater than the 25 percentile value but less than the 75^th^ percentile value); and *highest* quartile (all income values equal to or greater than 75% percentile value)]), level of education (primary, secondary, and tertiary), household population (< = 3 people, 4–6 people, >6 people), and hours of paid work (lowest quartile, median and highest quartile), walking status of last child (yes or no), occupation (not employed, semi-skilled, and skilled employment) were secondary independent (covariate) variables. Not all covariates were included in regression analyses.

Dependent Variables: The primary dependent variables were self-reported MSP (yes or no) lasting at least 60 minutes in continuous duration in the past two weeks for each of three anatomic regions—the lower back, the neck/shoulder, and the elbow/wrist/hand. Interviewers pointed to body diagrams (shaded body regions) and asked participants if they had experienced pain in any of these regions.

The secondary dependent variable was pain severity (no pain, mild pain, moderate pain, severe pain). Severity of self-reported pain was estimated using a numbered scale (from 0 to 10), with 0 representing ‘no pain’ and 10 representing the highest number for ‘severe pain (i.e., worst pain imaginable).’ Those with pain were categorized into mild, moderate, and severe pain with a value of 0 indicating no pain; 1–4 indicating mild pain, 5–7 indicating moderate pain; 8–10 indicating severe pain.

Sample Size Determination: With an alpha level of 5%, the statistical power of 80%, a minimum prevalence difference set at 10% and a prevalence ratio of 36% for water-stressed households, 26% prevalence in less water-stressed households [45], sample size was estimated to be 332 for women living in both groups of households. Assuming an attrition rate of 10%, the total sample size summed to 365 women.

### 2.3. Data analysis

Descriptive Analysis: Frequencies and percentages were derived for categorical variables, while means/standard deviations and medians/interquartile ranges were derived for continuous variables. Chi-square tests were used to assess any significant difference between demographic characteristics and the frequency of self-reported pain in each body region.

Logistic Regression models: Three logistic regression models were used to estimate the association between each of the six DWE factor scores (defined above) and a binary outcome of MSP in the back, the neck/shoulder, or the elbow/wrist/hand (yes or no) versus no pain in each region. Ordinal logit models were used to estimate associations between the DWE factor scores and MSP severity outcome that was categorized as four ordered responses (0 no pain; 1–4 mild pain, 5–7 moderate pain; 8–10 severe pain). First, multivariable logistic regression models including only the primary independent variables (DWE measures and hours of domestic work) were used to estimate the odds of MSP. Next, sociodemographic characteristics were included to adjust for potential confounding in the models. The same process was repeated for ordinal logistic regression models to estimate the odds of being in a higher level of pain versus a lower level of pain. Based on the Brant’s test results only the independent variables that satisfied the proportional odds assumption were included in ordinal models (*p*>0.05).

The measures of association were reported using odds ratios (OR) and 95% confidence interval (95% CI). All *p*-values < 0.05 were considered statistically significant. Multicollinearity among independent variables was examined within the regression models by (1) assessing the pairwise correlation between all variables and the variance inflation factor in regression models. Since all pairwise correlation coefficients < = 0.5 and VIFs were less than 2, There was no significant multicollinearity among variables [46]. Because the survey was interviewer-administered, completion rate across all variables except the DWEs was 98%. Details on how missingness was handled for factor items of each DWE measure were described in a previous study [32].

All data analyses were conducted using R statistical programs (R Core Team (2018). R: A language and environment for statistical computing. R Foundation for Statistical Computing, Vienna, Austria. URL https://www.R-project.org/.).

## 3. Results

### 3.1. Participant characteristics

Sociodemographic characteristics of the study sample have been previously described [36]. More than half of the 356 women (58%) experienced low back pain (LBP), 30% experienced neck/shoulder pain (NSP), and 29% experienced elbow/hand/wrist pain (EWHP) (Table 1). Approximately one-third (32%) of all women did not experience MSP in any body region, another one-third experienced pain in at least one of the anatomical regions, 20% experienced pain symptoms in at least two body regions, and 14% experienced pain symptoms in all three body regions. For the severity of LBP, out of 356 women, 43% experienced no pain, 26% rated LBP as mild, 12% rated LBP as moderate, and 19% rated LBP as severe. For NSP severity ratings, 71% experienced no pain, 14% rated pain as mild, 8% rated pain as moderate, and 7% rated pain as severe. For EWHP severity ratings, 73% did not experience pain, 14% rated EWHP as mild, 6% rated EHWP as moderate, and 8% rated EHWP as severe.

**Table 1:** Sociodemographic characteristics and musculoskeletal pain among 356 rural Nigerian women.

Variables	N	Low Back Pain		Neck/Shoulder Pain		Elbow/Wrist/Hand Pain		χ^2^ (p-values) ^a, b, c^
		Yes (%)	No (%)	Yes (%)	No (%)	Yes (%)	No (%)	
**Total**	356	209 (58)	149 (42)	105 (30)	251 (70)	104 (29)	252 (71)	
**Age [Mean ± SD] = 30.8 ± 6.5**								0.13; 0.11; 0.32
**17–25 years**	87	43 (20.7)	44 (29.5)	17 (16.2)	70 (27.9)	23 (22.1)	64 (25.3)	
**26 to 30 years**	103	68 (32.9)	35 (23.5)	36 (34.3)	67 (26.7)	32 (30.7)	71 (28.2)	
**31 to 35 years**	85	51 (24.6)	34 (22.8)	27 (25.7)	58 (23.1)	20 (19.2)	65 (25.8)	
**36 years and above**	81	45 (21.7)	36 (24.2)	25 (23.8)	56 (22.3)	29 (27.9)	52 (20.6)	
**Income/Month in Naira [Median ± IQR] = 15000 ± 35000 Naira**								0.81;0.85; 0.007
**Lowest Third [<10000]**	78	46 (22.2)	32 (21.5)	24 (22.9)	54 (21.5)	33 (31.7)	45 (17.9)	
**Median Third [10000 - < 30000]**	146	84 (40.6)	62 (41.6)	43(51.0)	103 (41.0)	42 (40.4)	104 (41.3)	
**Highest Third [≥30,000]**	132	77 (37.2)	55 (36.9)	38(36.2)	94 (37.5)	29 (27.9)	103 (40.9)	
**Education**								0.39; 0.14; 0.22
**Primary Education**	58	36 (17.4)	22 (14.8)	23 (21.9)	35 (13.9)	22 (21.1)	36 (14.3)	
**Secondary Education**	216	126 (60.9)	90 (60.4)	62(59.1)	154 (61.4)	57 (54.8)	159 (63.1)	
**Tertiary Education**	82	45 (21.7)	37 (24.8)	20 (19.0)	62 (24.7)	25 (24.0)	57 (22.6)	
**Household Size**								0.41; 0.02; 0.21
**0–3 people**	72	38 (18.4)	34 (22.8)	12 (11.4)	60 (23.9)	15 (14.4)	57 (22.6)	
**4–6 people**	174	107 (51.7)	67 (45.0)	56(53.3)	118 (47.0)	54 (51.9)	120 (47.6)	
**More than 6 people**	110	62 (30.0)	48 (32.2)	37(35.2)	73 (29.1)	35 (33.7)	75 (29.8)	
**Occupation**								0.13; 0.38; 0.65
**No paid work**	26	20 (9.6)	6 (4.0)	9 (8.6)	17 (6.7)	7 (6.7)	19 (7.5)	
**Semi-skilled job**	299	170 (82.2)	129 (86.6)	90 (85.7)	209 (83.3)	90 (86.5)	209 (82.9)	
**Skilled job**	31	17 (8.2)	14 (9.4)	6 (5.7)	25 (10.0)	7 (6.7)	24 (9.5)	
**Child Walking**								0.02; 0.75; 0.04
**Yes**	230	144 (69.6)	86 (57.7)	66 (62.9)	164 (65.3)	76 (73.1)	154 (61.1)	
**No**	126	63 (30.4)	63 (42.3)	39 (37.1)	87 (34.7)	28 (26.9)	98 (38.9)	
**Age of Last Child**								0.28; 0.49; 0.73
**Under 5 years**	178	109 (52.7)	69 (46.3)	56 (53.3)	129 (51.4)	54 (51.9)	128 (50.7)	
**Over 5 years**	178	98 (47.3)	80 (53.7)	59 (56.2)	122 (48.6)	50 (48.1)	124 (49.2)	
**Hours of Paid work/week [Mean± SD] = 40 ± 19.3 hours 0.23; 0.42; 0.54**								
**Lowest Third (< 35hr/week)**	112	72 (34.8)	40 (26.8)	38 (36.2)	74 (29.5)	30 (28.8)	82 (32.5)	
**Middle Third (35–50 hr/week)**	89	43(20.8)	39 (26.2)	24 (22.9)	58 (23.1)	22 (21.2)	60 (23.8)	
**Highest Third (>50 hr/week)**	162	92 (44.4)	70 (47.0)	43 (41.0)	119 (47.4)	52(50.0)	110 (43.7)	
**Hours of Domestic work/week [Mean ± SD] = 28.6 ± 10.4 Hours 0.05; 0.02; 0.68**								
**Lowest Third [< 23hr/week]**	117	58 (28.0)	59 (39.6)	23 (21.9)	94 (37.5)	31 (29.8)	86 (34.1)	
**Middle Third (23–30 hr/week)**	109	65 (31.4)	44 (29.5)	37 (35.2)	72 (28.7)	32 (30.8)	77 (30.5)	
**Highest Third (>30 hr/week)**	130	84 (40.6)	46 (30.0)	45 (42.9)	85 (33.9)	41 (39.4)	89 (35.3)	
**Main Water Source**								0.73; 0.73; 0.41
**Dug Well**	233	139 (67.1)	95 (63.8)	67 (63.8)	167 (66.5)	72 (69.2)	162 (64.3)	
**Piped water**	115	66 (31.9)	50 (33.6)	36 (34.3)	80 (31.9)	30 28.8)	86 (34.1)	
Rain and surface water**	8	--	--	--	--	--	--	

χ2 (p-values) a, b, c = chi-square p-values for association with Back a, Neck/shoulder b and Elbow/Wrist/Hand Pain c; ** = was excluded from analysis.

### 3.2. Frequency of MSP by sociodemographic conditions

MSP distribution across the categories of each sociodemographic variable was generally similar (Table 1). However, there were significant differences in the distributions of LBP, NSP, and EWHP across household size, income, and walking status of the last-born child. Women in larger households were more likely to report NP when compared to women from smaller households. Women who had children under-5 who were walking were more likely to report LBP and EHWP than those with children not yet walking. Women working long hours of domestic work were more likely to report NP when compared to shorter hours of work (Table 1).

### 3.3. Relationship between DWE measures, sociodemographic conditions, and MSP

The odds of experiencing LBP (OR = 4.26; 95% CI = 2.20–8.44; *p*<0.001) and NSP (OR = 4.44; 95% CI = 2.15–9.30; *p*<0.001) were significantly higher among women who perceived their domestic work responsibilities to be stressful (i.e., high adjusted ‘stress appraisal’ factor scores) after adjusting for important sociodemographic characteristics (Table 2). Being time-pressured and having lesser decision-making authority and control over domestic responsibilities, (i.e., ‘high demand and low control’ factor scores) was also associated with higher odds of experiencing LBP (OR = 2.30; 95% CI = 1.35–3.96; *p*<0.01) and NSP (OR = 1.36; 95% CI = 1.11–2.39; *p*<0.05). Insignificant associations were observed between ‘high’ demand and low control’ and EWHP (*p*<0.10). No statistically significant associations were detected between ‘water sourcing and carriage’, ‘experience of water scarcity’, ‘social support’, and ‘frequency of domestic tasks’ and measures of LBP, NSP, and EWHP. Concerning sociodemographic conditions, the odds of experiencing EWHP were significantly higher among women in the lowest income group (OR = 3.73; 95% CI = 1.85–7.38; *p*<0.001) compared to those in the highest income group. The odds of experiencing LBP (OR = 2.61; 95% CI = 1.27–5.48; *p*<0.01) and NSP (OR = 2.26; 95% CI = 1.13–5.07; *p*<0.05) were higher among women in the mid-reproductive age group (26–30 years) compared to those in the youngest age group (ages 18–25). The odds of NSP were higher among women with primary education (OR = 2.34; 95% CI = 1.14–5.47; *p*<0.05) than those with tertiary education. There was no significant relationship between education and either LBP or EWHP.

**Table 2:** Odds of the presence versus absence of the association between DWE measures, sociodemographic characteristics, and MSP.

Predictor Variables	Low Back Pain		Neck/Shoulder Pain		Elbow/Hand/Wrist Pain	
	OR [95% CI]	p-value	OR [95% CI]	p-value	OR [95% CI]	p-value
Frequency of Domestic Tasks	0.85 [0.41–1.77]	0.14	0.84 [0.33–2.11]	0.70	0.73 [0.38–1.59]	0.33
Water Sourcing & Carriage	1.12 [0.96–1.32]	0.09	1.11 [0.94–1.62]	0.16	1.20 [0.83–1.76]	0.23
Water Scarcity	0.80 [0.57–1.12]	0.18	0.95 [0.66–1.35]	0.58	0.61 [0.42–1.29]	0.13
Stress Appraisal	4.26 [2.20–8.44]	>0.001	4.44 [2.15–9.30]	>0.001	1.50 [1.04–3.02]	0.06
Demand and Control	2.30 [1.35–3.96]	0.002	1.36 [1.11–2.39]	0.04	1.55 [0.98–2.73)	0.07
Support	0.70 [0.45–1.13]	0.142	0.70 [0.40–1.10]	0.19	0.95 [0.59–1.72]	0.90
Hours of Domestic Work: Lower Quartile (Ref)						
Middle Quartile	1.39 [0.77–2.49]	0.27	1.70 [0.89–3.28]	0.11	1.09 [0.58–2.05]	0.78
Upper Quartile	1.70 [0.96–3.03]	0.06	1.60 [0.86–3.03]	0.14	1.14 [0.62–2.12]	0.65
Age: 18–25 years (Ref)						
26–30 years	2.61 [1.27–5.48]	0.009	2.26 [1.13–5.07]	0.044	1.29 [0.60–2.79]	0.51
31–35 years	2.11 [1.04–4.77]	0.052	2.00 [0.85–4.82]	0.11	0.75 [0.35–1.78]	0.51
> = 36 years	1.68 [0.73–3.91]	0.22	1.81 [0.77–4.68]	0.18	1.54 [0.65–3.73]	0.33
Age of Youngest Child: < 5 years (Ref)						
Under 5 years	1.29 [0.74–2.25]	0.36	0.98 [0.55–1.73]	0.93	1.04 [0.59–1.85]	0.65
Household Size						
4–6 people	1.21 [0.59–2.52]	0.59	2.00 [0.84–4.82]	0.08	2.09 [1.11–4.75]	0.056
>6 people	0.94 [0.48–2.24]	0.88	1.86 [0.86–6.05]	0.11	2.23 [0.95–5.87]	0.091
Income: Upper Quartile (Ref)						
Middle Quartile	1.38 [0.79–2.41]	0.26	1.04 [0.55–2.30]	0.89	1.81 [0.98–3.41]	0.052
Lower Quartile	1.51 [0.78–2.97]	0.22	1.13 [0.57–1.87]	0.73	3.73 [1.85–7.38]	> 0.001
Education: Tertiary (Ref)						
Secondary	1.49 [0.82–2.74]	0.11	1.48 [0.78–2.91]	0.24	0.70 [0.37–1.34]	0.28
Primary	1.89 [0.96–4.22]	0.082	2.34 [1.14–5.40]	0.037	1.28 [0.57–2.86]	0.45
Hours of Paid Work: Lower Quartile (Ref)						
Middle Quartile	0.57 [0.30–1.10]	0.10	0.89 [0.44–1.76]	0.74	1.00 [0.42–2.03]	0.89
Upper Quartile	0.69 [0.40–1.20]	0.19	0.72 [0.49–1.31]	0.29	1.47 [0.82–2.69]	0.20

OR = Odds ratio; CI = Confidence Interval, Ref = reference group.

### 3.4. Relationship between DWE measures, sociodemographic conditions, and MSP severity

**Fig 1:**
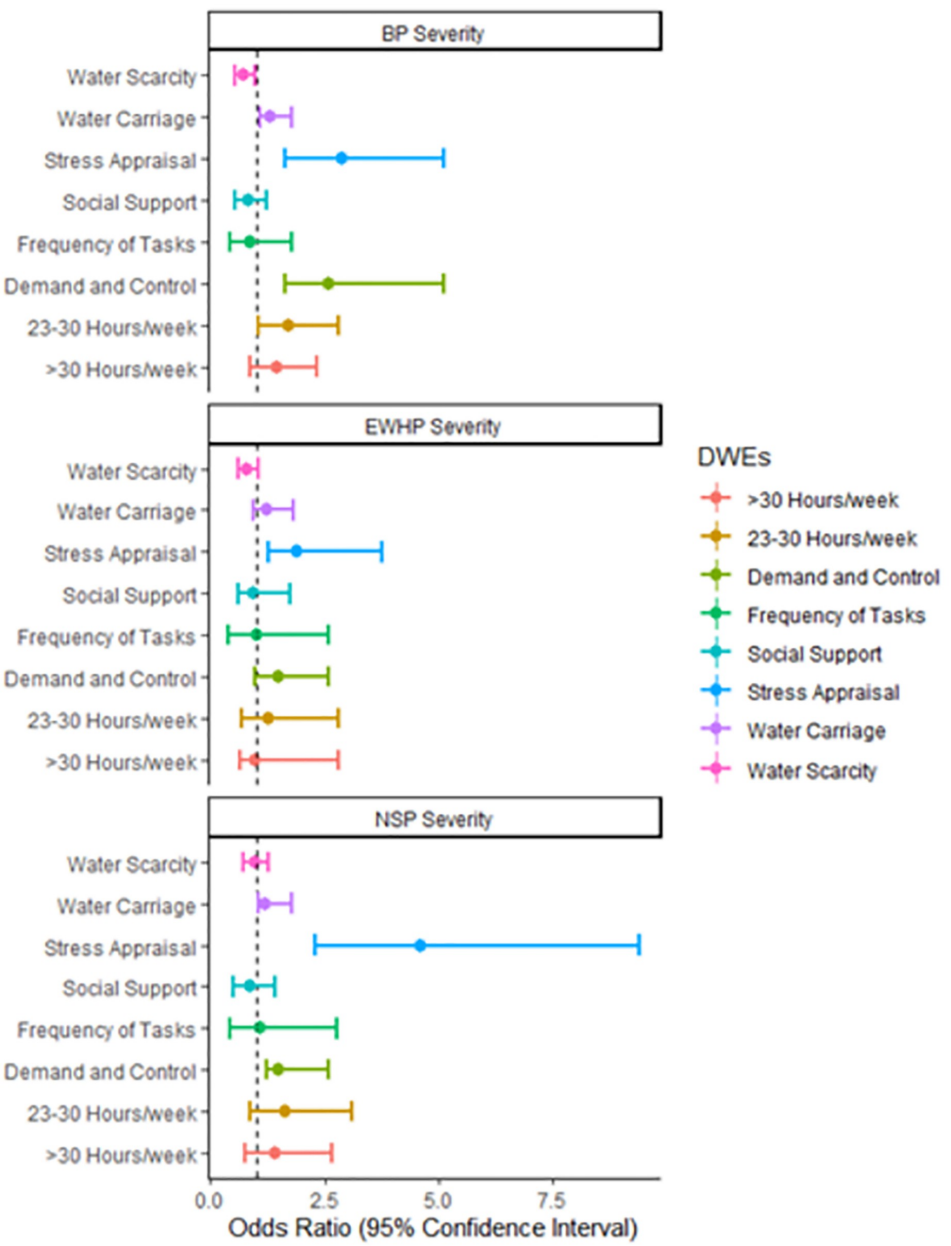
Forest Plot showing the adjusted association between severity of musculoskeletal pain (LBP, NSP, and EHWP) and domestic work experience measures. The dotted line represents the reference level (Odds ratio = 1).

The odds of having more severe versus less severe LBP (OR = 2.88; 95% CI = 1.64–5.11; *p* < 0.001), NSP (OR = 4.58; 95% CI = 2.29–9.40; *p* < 0.001), and EHWP ratings (OR = 1.88; 95% CI = 1.26–3.77; *p* < 0.05) were significantly higher for each one standard deviation (SD) increase in the stress appraisal factor score (Fig 1; S1 Table). Higher ‘demand and control’ factor scores were also significantly associated with higher odds of more severe LBP (OR = 2.58; 95% CI = 1.64–4.09; *p* < 0.001) and NSP ratings (OR = 1.49; 95% CI = 1.24–2.58 *p* < 0.001) and trended towards similar associations with EWHP (OR = 1.48; 95% CI = 0.98–2.59; *p* < 0.05). A high ‘water sourcing & carriage’ factor score was associated with more severe LBP (OR = 1.31; 95% CI = 1.09–1.79; *p* < 0.05) and NSP ratings (OR = 1.20; 95% CI = 1.08–1.76; *p* < 0.05) and trended towards similar associations with EWHP (OR = 1.23; 95% CI = 0.94–1.81; *p* = 0.072). Spending between 23–30 hours/week (middle quartile) on domestic work (OR = 1.72; 95% CI = 1.25–2.8; *p* < 0.05) was significantly associated with more severe pain versus those in lower quartile. There was no significant relationship between ‘social support’, ‘experience of water scarcity’, and ‘frequency of domestic tasks’ with LBP, NSP, and EWHP severity ratings (Fig 1; S1 Table). The odds of having more severe LBP were significantly higher among women ages 26–30 years (OR = 2.12; 95% CI = 1.16–3.95; *p* < 0.05); and women ages 31–35 years (OR = 2.16; 95% CI = 1.07–4.35; *p* < 0.05) compared to women between 18–25 years; and those who have only primary education (OR = 1.89; 95% CI = 1.15–3.79; *p* < 0.05) compared to those with tertiary education. The odds of having more severe NSP were only significantly higher among women with only primary education (OR = 2.24; 95% CI = 1.13–5.04; *p* < 0.05) compared to those with tertiary education. More severe EHWP odds were only significantly higher among women in the lowest income quartile (OR = 3.17; 95% CI = 1.59–6.38; *p* <0.001) compared to those in the highest income quartile.

## 4. Discussion

This research examined whether recently developed DWE measures representing physical, psychosocial, and social conditions for women in rural Nigeria are associated with the presence and severity of MSP after adjusting for important sociodemographic factors. Multiple factors, especially those representing physical (water sourcing and carriage) and psychosocial factors (stress appraisal and demand-control), had consistently strong relationships with the presence and severity of pain in LBP, NSP, and EWHP as indicated by the direction of the relationship and the significance of the effect size. This consistency bolsters the conclusion that these are real and not spurious associations between DWEs and MS. Thus, psychosocial, and physical factors (psychosocial stress from domestic work demands, physical stress of water carriage) of domestic work were independently linked to MSP among rural, low-income Nigerian women living in water-insecure communities.

The 2-month prevalence of LBP (58%) in this study was higher than the 52.7% median 12-month prevalence estimate derived from a systematic review of 12 studies among Nigerian adults [47]. Prevalence of MSP (LBP, NSP Prevalence of MSP (LBP, NSP and EWHP) was similar to the 7-day prevalence estimates (36% in South Africa; 57.3% in Ghana; 54.3% in Vietnam) in a three-country study among adults with a history of water carriage (40). The 2-month prevalence of MSP pain in this study (68%) (i.e., experiencing pain symptoms in one or more anatomical regions- back, neck/shoulder, elbow/hand/wrist (LBP and EWHP) was slightly lower but similar to those reported among homemakers in rural India (70–78% 12-month prevalence) [48] and among studies explicitly addressing the relationship with domestic work exposures and MSP among rural women [45,49]. The estimated prevalence of multi-region or co-occurring pain was also similar to the estimates from other studies [38,45,48]

### 4.1. Physical factors of domestic work experiences and musculoskeletal pain

There was no significant association between the frequency of domestic tasks and experience of MSP. Our finding is in line with previous studies [50–52], which found insignificant associations between frequent engagement in domestic work and MSP (LBP, NSP) among women. Hours of domestic work were associated with more severe LBP (but not NSP and EWHP); the more hours women worked, the greater the chances of pain, which agrees with the conclusion from previous studies [53,54].

Experience of water scarcity was not significantly associated with the presence and severity of MSP among this population. This could be because communal water sharing buffers the adverse experiences s of water scarcity despite women not having on-plot water services [36]. However, the work to move water into the household, as measured by high water sourcing and carriage scores, was associated with a higher odd of LBP and NSP. Women who spent long-time procuring water or were frequently responsible for water carriage and collection trips had increased risks of experiencing severe LBP or NSP compared to women who have on-plot water services.

Although women experience physical stress and MSP while engaged in water sourcing and collection labor, they may not have a negative cognitive experience of water scarcity (i.e., worry, frustration, and anger) because of neighborhood water sharing [55]. A multi-site cross sectional study carried out in Ghana, South Africa, and Vietnam found a strong positive and significant association between current or history of water carriage and self-reported pain in the upper back and hands, but a negative association in the neck or lower back [38]. This supports our results that history of water carriage (typified by being responsible for water sourcing, head loading, and making frequent and long water trips) was associated with self-reported presence and severity of LBP and NSP [38]. Typically, access to water has been addressed in terms of distance to water and time spent collecting water [56]. The physical burden of work, including the risk of MSP, can be inferred from such measures especially for those making long trips; however, spending less time (such as 10 minutes) on water carriage does not preclude the absence of MSP risks. For example, dug wells are readily located within compounds or nearby in rural west-African communities. The water must be fetched and transported into the dwelling, even if the water-trip takes less than 5 minutes a day. Thus, including water sourcing and collection measures—such as frequency of water trips, number of trips per day/collection period, and frequency of head loading—are important in LMICs to understand the dimensions of water insecurity and may be useful in further highlighting the impact of access to water on health [55,57]. In Africa, regular head loading, and water carriage has been linked to the development of chronic MSDs such as cervical spondylosis and intervertebral disc degeneration, which may result in severe trauma and disability [39,58]. Understanding and assessing the MSD risk attributed to water collection labor especially among rural women is crucial because of their increased vulnerability by virtue of being disproportionately exposed to other MSD risk factors (e.g., poor nutrition, psychosocial stress, poor access to health care and labor-saving infrastructure).

While water collection is a well-understood cause of psychosocial stress and MSP, it is one of many domestic tasks that can contribute to these outcomes. The physical time and effort spent on other domestic tasks was not directly measured in this survey but is likely measured through the aggregate indicators of hours spent on DWE and psychosocial factors.

### 4.2. Psychosocial factors and musculoskeletal pain

Psychological appraisal of domestic work responsibilities as stressful, or ‘stress appraisal’, was consistently and strongly associated with high odds of MSP. Psychosocial stress results from the interaction between external demands, such as work responsibilities, and the woman’s cognitive appraisal of her capacity to cope with those demands. Our previous study demonstrated the link between strenuous water collection labor and increased psychosocial stress (38), which was significantly associated with MSP in this paper. Manual loading (physical stress) combined with psychosocial stress may induce muscular tension and MSP. Psychologically stressful jobs with moderate to low physical work significantly increase the risk of MSDs because of the additional muscle tension caused by physiological stress [59–61]. Our result is consistent with other literature that found a positive association between psychological distress from non-water-related domestic work responsibilities and MSP among women [62–64].

Women’s appraisal of domestic work stress as ‘stressful’ may be aggravated by stress from paid work responsibilities, so it is important to consider the total life experience of a woman when measuring life stressors [27,59,60]. Realistically, it may be difficult to separate the adverse impacts of stress from domestic work from those from paid work.

High demand and low control scores were associated with increased odds of experiencing MSP. Performing domestic work under time pressure, especially in the morning when women are getting their family ready for daily activities, may increase the work speed and the resulting forces exerted on domestic tasks, especially for women with young children [65]. Also, low job control or low work autonomy has been found to shorten the rest and recovery phases that are needed to reduce musculoskeletal strain [50,66]. Thus, low job control and time pressure in domestic work may increase the risk of pain in the neck and upper limbs just as in paid work [67]. However, the influence of psychosocial factors on the risk of MSP in domestic work may not be as severe as in paid work.

### 4.3. Social factors of domestic work experiences and musculoskeletal pain

There was no significant relationship between social support and MSP, although the odds of MSP were lower among women reporting social support. Poor social support reduces the opportunities for responsibility-sharing and division of labor. It increases the risk of MSDs, while high social support (especially instrumental support) reduces the risk for MSDs [67,68]. Further, husband’s involvement with household responsibilities was strongly associated with positive psychosocial health of women [69]. Social support is integral to improving women’s health and behaviors [70]. Social support indicators, such as social connectedness and strong network ties, have been associated with psychological resilience among survivors of intimate partner violence [71] and improved food security and mental health [72] in Africa. In addition, social capital, defined as communal collective efficacy and social cohesion, buffered the effect of inadequate access to water and sanitation on birth outcomes (low birth weight) in India [73]. Also, household and neighborhood water sharing, a form of instrumental support, are adaptive strategies that reduce the physical demands of water collection labor, consequently reducing the risk of MSP [55,57]. The lack of a significant association between social support and MSP in our study may have been because social support was defined as instrumental support from family or household members. This definition may not have accounted for the full meaning of social support because it excluded instrumental support from communal or neighborhood networks, and emotional support from family members.

### 4.4. Sociodemographic factors of domestic work experiences and musculoskeletal pain

Most sociodemographic variables such as income, education, household size, child’s age, and paid work hours were either insignificant or marginally associated with MSP. This result may be due to the homogenous sociodemographic profile of women in the selected communities. For example, most women were from low-income families (median income less than minimum wage) and with secondary education. Increased household size, age, and the number of young children probably increased the number of hours women spent on unpaid work. However, these factors did not result in significantly higher odds of MSP as in other studies [49,54].

There was a significant relationship between being in a younger reproductive age group and having MSP. Compared to women between the ages of 18–25, women between 26–30 years were significantly more likely to experience MSP (particularly in the back and neck/shoulder). In addition, the odds of MSP were higher in the 26–30 age group when compared to older (36 years and above) women. This finding could be because women within those active reproductive age groups are more likely to be responsible for domestic work if they live in compound/extended family households, and they may be more likely to have more young children than older women [74]. Our result contrasted with others that found that increased age was associated with increased MSP [37,49,62]. However, the average age for older women in these other studies was much higher than that of older women in our study. This study’s mean age was lower than other studies because our study population was restricted to premenopausal women, so age-attributed, physiological effects that may result in increased MSD risk, was attenuated.

### 4.5. Strengths and limitations

This study examined the relationship between MSP and DWE by utilizing a multi-dimensional measure of exposure that captures the DWEs of women in rural communities in LMICs. We adjusted for the effects of important covariates while estimating the association between MSP and DWE. We used body diagrams in the questionnaire and a pain-rating scale to ensure that women understood how to rate pain accurately, the degree of pain and ascertain the location of the pain. This approach enhanced reliability of reporting and the validity of our pain measures.

Because of our cross-sectional design, causal association between DWEs and MSP cannot be fully established We could not determine whether the exposure was an antecedent of the outcome or vice versa. The risk of MSD may be over or underestimated in this study because women who experience pain may be more cognitively aware of the factors, they think influenced their pain. Also, we could not accurately assess self-reported biomechanical risk factors of domestic work, such as awkward postures, repetitive movement, and bending and squatting through survey [36]. Forthcoming studies will report on these aspects of posture, movement, and physical activity during domestic work.

Similarly, our definition of social support did not fully capture some aspects of social support (emotional support, support from outside the family). Therefore, our DWE measure may not fully capture the physical factors/demand of domestic work. Data was collected from rural, low-resource communities, which limits our ability to generalize findings to other populations of women in Nigeria and beyond. Our study was not adequately powered to allow us to assess EHWP, including the interactions between latent factors (physical, psychosocial, and social domains), so we did not include these interactions in our logistic models.

### 4.6. Conclusion

The study contributes critical evidence on the health effects of domestic work among women in rural Sub-Saharan Africa. Our evidence supports the hypothesis that strenuous domestic work increases the risk of MSP among rural Nigerian women. If left unaddressed, chronic MSP can become debilitating and progress to MSD (the most common contributor to disability globally). Both physical (water sourcing and carriage) and psychosocial factors (demand/control and stress appraisal) contribute to women’s experience of MSP. Through understanding the impact of domestic work on women’s musculoskeletal health, policymakers and researchers can use the information from this study to advocate for improved behavioral, social, and infrastructure interventions that can potentially improve gender equality in the assumption of domestic roles and, consequently, women’s health.

More extensive prospective studies are needed to estimate the association between DWEs and MSP. Future research should focus on including other measures (e.g., positive coping strategies, emotional and neighborhood support, social capital) that could buffer the adverse impact of domestic work on MSP. Examining this association among rural women at different life stages (i.e., newly married, pregnant, middle-aged, and post-menopausal women) would be useful to understanding exposure-outcome variations.

## Supporting information

S1 Table Odds of severe (8–10-point pain rating) versus less severe (5–7 and 1–4 pain ratings) of the association between DWE measures, sociodemographic characteristics, and Musculoskeletal Pain (MSP). (DOCX)

S1 Dataset (CSV)
