# Supplementary material for: Assessing the relationship between domestic work experience and musculoskeletal health among rural Nigerian women
Source: PLoS One. 2022 Dec 13;17(12):e0276380. doi: 10.1371/journal.pone.0276380 (PMC9747006; doi:10.1371/journal.pone.0276380)
Supplement: S1 Table — (DOCX) [file pone.0276380.s001.docx]

| S1 Table . Odds of severe (8–10-point pain rating) versus less severe (5-7 and 1-4 pain ratings) of the association between DWE measures, sociodemographic characteristics, and MSP | | | | | | |
| --- | --- | --- | --- | --- | --- | --- |
| Predictor Variables | Back Pain | | Neck/Shoulder Pain | | Elbow/Hand/Wrist Pain | |
|  | OR [95% CI] | p-value | OR [95% CI] | p-value | OR [95% CI] | p-value |
| Frequency of Domestic Tasks | 0.85 [0.41-1.77] | 0.72 | 1.1 [0.44-2.78] | 0.82 | 1.00 [0.40-2.57] | 0.87 |
| Water Sourcing & Carriage | 1.31 [1.09-1.79] | 0.044 | 1.20 [1.05-1.78] | 0.047 | 1.23 [0.94-1.81] | 0.072 |
| Water Scarcity | 0.73 [0.54-0.98] | 0.05 | 0.98 [0.73-1.26] | 0.53 | 0.78 [0.59-1.05] | 0.068 |
| Stress Appraisal | 2.88 [1.64-5.11] | >0.001 | 4.58 [2.29-9.4] | >0.001 | 1.88 [1.26-3.77] | 0.03 |
| Demand and Control | 2.58 [1.64-4.09] | >0.001 | 1.49 [1.24-2.58] | 0.02 | 1.48 [0.98-2.59] | 0.065 |
| Support | 0.81 [0.53-1.24] | 0.33 | 0.88 [0.50-1.40] | 0.47 | 0.95 [0.59-1.73] | 0.89 |
| Hours of Domestic Work: Lower Quartile (Ref) | | | | | | |
| Middle Quartile | 1.72 [1.05-2.8] | 0.038 | 1.63 [0.87-3.08] | 0.13 | 1.26 [0.67-2.35] | 0.46 |
| Upper Quartile | 1.45 [0.88-2.34] | 0.14 | 1.41 [0.77 -2.64] | 0.27 | 0.99 [0.63-2.79] | 0.94 |
| Age: 18-25 years (Ref) | | | | | | |
| 26-30 years | 2.12 [1.16-3.95] | 0.015 | 2.09 [1.02-4.5] | 0.057 | 1.32 [0.63-2.79] | 0.47 |
| 31-35 years | 2.16 [1.07-4.35] | 0.032 | 1.69 [0.72-3.96] | 0.23 | 0.88 [0.37-2.07] | 0.76 |
| >= 36 years | 1.96 [0.93-4.17] | 0.065 | 1.81 [0.74-4.45] | 0.19 | 1.40 [0.59-3.35] | 0.44 |
| Age of Youngest Child: < 5 years (Ref) | | | | | | |
| Under 5 years | 1.06 [0.75-1.70] | 0.83 | 0.96 [0.55-1.68] | 0.89 | 1.04 [0.59-1.85] | 0.88 |
| Household Size |  |  |  |  |  |  |
| 4-6 people | 1.39 [0.74-2.64] | 0.31 | 1.70 [0.77-3.88] | 0.19 | 1.40 [0.81-4.05] | 0.15 |
| >6 people | 1.20 [0.52 -2.09] | 0.64 | 2.14 [0.84-5.60] | 0.11 | 2.31 [0.90-6.05] | 0.08 |
| Income: Upper Quartile (Ref) | | | | | | |
| Middle Quartile | 1.23 [0.76-2.01] | 0.35 | 1.12 [0.56-2.25] | 0.73 | 1.58 [0.85-2.95] | 0.12 |
| Lower Quartile | 1.31 [0.73-2.32] | 0.39 | 1.03 [0.58-1.84] | 0.90 | 3.17 [1.59-6.38] | 0.001 |
| Education: Tertiary (Ref) | | | | | | |
| Secondary | 1.69 [1.08-2.89] | 0.046 | 1.38 [0.72-2.70] | 0.33 | 0.75 [0.4-1.45] | 0.39 |
| Primary | 1.89 [1.15-3.79] | 0.041 | 2.24 [1.13-5.04] | 0.04 | 1.38 [0.6-3.08] | 0.42 |
| Hours of Paid Work: Lower Quartile (Ref) | | | | | | |
| Middle Quartile | 0.60 [0.34-1.08] | 0.09 | 0.92 [0.47-1.79] | 0.81 | 0.86 [0.42-1.76] | 0.69 |
| Upper Quartile | 0.70 [0.43-1.13] | 0.14 | 0.69 [0.38-1.23] | 0.21 | 1.26 [0.71-2.31] | 0.46 |

OR= Odds ratio; CI= Confidence Interval, Ref= reference group
